# Sister Olive: A Hospital Romance

**Published:** 1886-12-04

**Authors:** W. J. Eccott


### Sister Olive: a Hospital Romance.

By W. J. ECCOTT.

### CHAPTER I.

Two or three students were coming up the stairs of "The Infirmary" at Cottingham with the view of going into the "Theatre" to witness an operation, which had just begun. As they reached the landing and approached the opening door the mingled fumes of carbolic acid and ether made themselves pungently felt, and the tiers of students, all eagerly watching the spectacle proceeding below, could be seen. Two of them came out; one looking deathly pale, and leaning on the other.

" Hullo, Leslie !" said one of the new comers, named Thomson. " First operation, eh ! Never seen blood before ?"

" I do feel rather faint," returned Leslie almost in a whisper—his lips refusing to perform their office. " A little fresh air and I shall be all right."

" By jove, man ! You do look jolly bad," said another. " Doesn't he, Archie !"

The student addressed was a well built, curly headed young man, of about twenty-two, with rather heavy eyebrows, and a cautious look about his eyes.

" Oh ! It's nothing !" he said, with something of contempt in his tone. " You'll get used to it. It's more the smell of the spray than anything. Are you fellows going in ?"

" Is it anything particular ?" Thomson inquired of Leslie's companion. " Who's there ?"

" It's only an amputation ! Temston's operating. Sandy and Riordan are there, and Grant."

" Grant's leaving, isn't he ?" said Leslie, who was fast recovering.

" So I hear," said Thomson. " Nice fellow, Grant ; awfully sorry he's leaving."

" Queer fellow, I think," said Archie Tenison. " Puts it on a good bit. He's tried to spite me once or twice."

" Only your fancy," said one of the others. " I think he's a decent fellow, and a clever one too, but there's something of the cynic about you, Archie. You've got something up against him. But what's he going to do ? Set up in private practice."

" Yes ! I suppose so," said Archie, in an off-hand fashion.

They were standing at the head of the stairs, and did not notice one of the " sisters" come up, until, at the sound of a soft voice, " Excuse me ! gentlemen," they move aside and said in tones of politeness and respect, " Good morning ! Sister Olive !"

Archie Tenison blushed slightly, as she returned their salute and passed into an adjoining ward. Even in her gown of simple hue, relieved only by her white apron, collar, and the cap which confined, but scarcely hid, the coils of glossy hair, she was a woman to look twice at. Her well-cut features and gray eyes, which seemed of themselves to speak volumes, made her face one little likely to be forgotten.

" What ! Archie, the cynic, blushing ? " exclaimed Thompson.

" Archie Tenison ! Gone on Sister Olive ! " said another, laughing.

" Bosh ! " said Archie, colouring again, and then, somewhat sternly—" I'll thank you men not to make Sister Olive a subject for chaff, as if she was a barmaid at 'The Feathers.' "

" Oh, you needn't cut up rough," said Thomson. " Of course, it's all right. Still it's very strange that you're so often to be found in her ward. And she seems to be better supplied with flowers than the others."

" That beggar Grant gives them to her then," exclaimed Archie, somewhat bitterly.

" Oh ! That's how the land lies, does it ? " said Leslie. " Then you haven't got the preserves all to yourself ? "

Archie made no reply. Looking over the stairs, he exclaimed, "Here's Scuffy and some of the men. I'm going to hear him take a case in Ward 14."

"Sister Olive's ward, of course!" sneered Thomson. "Let's all go, then. What is it?"

"Enteric fever!" said Archie. "Just been brought in. It was only yesterday or so."

Just then Professor Lanscuph, familiarly known to the students as "Scuffy," came up the stairs, followed by a throng of students, and the group joined with them and went into the ward over which Sister Olive had charge.

A slight odour of antiseptics pervaded the room, round which the beds were ranged at wide intervals along the walls. Down the centre of the ward were long tables, bearing here and there a few vases of flowers, and two or three large bottles containing either antiseptic fluids or sponges soaking in them. Upon the walls were a few pictures and texts. All was clean to a degree, and quiet save for the occasional incoherent mutterings of a restless patient.

As the Professor entered, most of the patients looked up eagerly.

On the bed, around which the Professor and his class gathered, lay a man of apparently strong frame, but with cheeks wasted and sallow, and eyes sunken. One of the nurses, under Sister Olive's directions, had just been sponging the patient's mouth and nostrils, and drew away as the Professor approached. The occasional incoherent phrases, which dropped half spoken rather than uttered from his lips, bespoke the early stages of delirium. Over his bed, on a printed form, was written: "Name—John Hargen; occupation, engineer; age, 32." He had sickened on board ship, so Sister Olive told the Professor, and had only landed to come into the hospital. He had been in two days. Little else had been done but to prevent him from taking cold, administer cooling draughts, and keep mouth and nostrils clean.

The Professor made a careful examination of the patient, adding various comments, which many of the students noted on paper, of the nature and symptoms of the disease, describing its best treatment and giving sundry directions to Sister Olive as to the course to be pursued.

While he was talking, Hamelin Grant, the house physician had come in. He was a man not over thirty, with a fine studious face, which wore a grave expression as he stood listening intently to the Professor, who occasionally spoke with him about the case. He seemed greatly interested in it.

When the class went out, Grant stayed behind a few minutes and said to Sister Olive, in a tone which betrayed some nervousness: "Can you come down to my room in the course of half an hour or so? I am leaving to-day, you know, and I want to talk to you a little."

A look of sadness passed over her features as she said: "I'm so sorry you're leaving, Doctor Grant. And the patients, I know, like you very much. It will be a great loss."

"Not at all, Sister Olive! They've a good man coming in my place, so I hear!"

"Who is he?" she asked.

"A Bernard Lumley!"

"Bernard Lumley!" she exclaimed in a tone in which surprise and anger mingled.

"Yes! Do you know him? You look as if he wouldn't please you?" said Grant.

"Oh! I know something of him," she said, half turning her head. "Very well, Doctor Grant. I'll come down in half an hour!" and she turned to a patient.

In due time a knock came at the house-physician's door, and Sister Olive entered.

"Thank you for coming down," he began. "Please sit down. I wished very much to speak to you before I left. I may not get another chance." Then he went on rather nervously: "We've been very good friends since we first met. Haven't we?"

"Yes!" said Sister Olive earnestly, and looking straight at him. "And we still shall be good friends, shall we not?"

Grant took one or two paces up and down, knitting his brows slightly, and then turned again to her—

"Will you be offended if I speak as plainly to you as I would were you my own sister?"

"Between true friends, plain speaking can never give offence," she said.

"Is there any tacit engagement existing between you and Archie Tenison, the student?" Grant asked abruptly.

Sister Olive blushed and looked down.

"No!" she said; there is nothing. Mr. Tenison has paid me many little attentions, been very kind to me. But there is no engagement, tacit or otherwise, between us."

"Then I may speak freely," said Grant with emotion, as he looked straight into her face. She was confused, and turned away her face as he went on earnestly.

"You have known me as a somewhat grave man, who values his life work above everything. I have been in all crises of my life self-reliant, trusting to my own energies to overcome difficulties and to achieve success. But there are occasions in a man's life when he feels—the want of sympathy. I mean something more than the sympathy of the world in general, or of that little corner of it which is more especially his own. I mean the loving sympathy of one heart to whom all his aspirations, all his cares, may be confided. With me that time has come. And more especially now that I am exchanging the bustling life of the Infirmary, and the society of my friends here, for the lonely path of private practice. For a long time past I have had this feeling. I have reviewed it carefully. It is no boyish fancy or fleeting emotion, but a deep lasting passion which has become part of my very being. It has centred itself in one object. I must speak out Olive. It is yourself."

His tones had gathered strength as he went on. The earnest emotion in them carried conviction of the truth of his words. He paused a moment. Sister Olive still looking down, he went on:

"Olive! dare I ask you to let me hope that you will be the helpmeet, the sympathiser, the woman who is to make my life useful, my wife?"

Olive raised her head. Her grey eyes looked sad. There was a slight quiver about her lips. Then she spoke in tones which expressed a heartfelt sympathy which went straight to Grant's heart.

"Hamelin, I feel proud, honoured, that you of all men should have made this offer; should have singled out me as the fittest companion of your life. I feel how high a work this is of yours. I know how well you have wrought here in this hospital. I know how nobly you will pursue that course in after life. Believe me, I should be proud to share it, but Hamelin, I cannot promise anything."

Grant's face, which had been lighted up with so much hope, became overcast, and, in a voice which seemed as if something were sticking in his throat, he said:

"Perhaps I've been too hasty. Pardon me, if I have."

"No; not too hasty, Hamelin! No! We ought to know each other. The daily contact, the unison in work for months together, must have taught us to know each other."

"Can you, can you hold out any hope, then?" he faltered.

Sister Olive shook her head, too overcome to trust herself to speak.

"What are the difficulties? Can they be overcome? Don't say that my whole life is to be clouded over by one over-mastering regret, one which will cripple half my powers," he pleaded.

"Hamelin; I cannot even hold out hope. You know nothing of my history, nothing of my family," she said.

"Nothing of your history!" Grant exclaimed. "Before you came here—no! But of long months of unwearying patience, of unflinching sympathy and zeal for others—Is that nothing? I do not ask questions about antecedents, about family. It is yourself, as you are now! Whatever your motives were in taking up the work you have done, you have proved yourself worthy of the work, worthy of the noblest future. But I do not hold out any brilliant prospect. To be the wife of a hard-working general practitioner is no sufficient inducement to hold out. But tell what the difficulties are!"

"Hamelin;" she answered. "Perhaps you will know them some day. But now I can only tell you that my life is clouded over. At present I cannot lift the veil. It may be possible some day. Until it is, I cannot, for your sake, consent in any way to let your lot be linked with mine, lest you should suffer from taking an irrevocable step, and I could never forgive myself. No, Hamelin! Do not press me! It would only pain me more. You must learn to forget me in this connection. Find in your work the healing for what I cannot but feel will be a wound for many a day. You will always be a dear friend. May the cloud that rests upon me never come between us as friends. More I cannot say. You may think me strange. If I could say more now I would. Hamelin, for the present—good-bye!" and she placed both hands in his.

"Well, Olive," said Grant, in a husky voice, "this is a great grief to me. But I will not try to penetrate your secret. I am not going to give up all hope. Nevertheless, it will be hard work for a long time to do my best with this disappointment hanging over me. Come, you will promise me this—that if ever you need a friend, one who will do for you what father, brother, husband might, you will send for me. Olive, Good-bye!"

(*To be continued.*)

